# Butyl­bis[μ-4-(2,4,6-trimethyl­phenyl­amino)pent-3-en-2-onato][4-(2,4,6-trimethyl­phenyl­amino)pent-3-en-2-onato]dimagnesium

**DOI:** 10.1107/S1600536809033327

**Published:** 2009-08-29

**Authors:** René T. Boeré, Twyla Gietz

**Affiliations:** a Department of Chemistry and Biochemistry, The University of Lethbridge, Lethbridge, AB, Canada T1K 3M4

## Abstract

The structure of the title compound, [Mg_2_(C_4_H_9_)(C_14_H_18_NO)_3_], contains two Mg atoms bridged by two μ_2_-O atoms from two of the three ketiminate ligands, while the third ketiminate is strictly chelating to one of the Mg atoms, which is thereby five-coordinate. In place of a chelating ligand, the second Mg atom is ligated by a single terminal *n*-butyl group and thus is four-coordinate. This is, so far, the only structurally characterized mixed magnesium ketiminate–alkyl cluster. The geometry at the first Mg atom is close to trigonal-bipyramidal with one chelating and one bridging O atom in the axial positions and two chelating N and one bridging O atom in the equatorial positions. The geometry at the second Mg atom is very distorted from tetra­hedral, with an O—Mg—C angle of 131.0 (1)°.

## Related literature

For structures of the other known magnesium–ketiminate complexes, see: pioneering study (Corraza *et al.*, 1988 ▶); application to chemical vapour deposition (Matthews *et al.*, 2000 ▶, 2005 ▶; Ouattara *et al.*, 2005 ▶; Sedai *et al.*, 2008 ▶); applications in catalysis (Lee *et al.*, 2007 ▶; Tang *et al.*, 2007 ▶). For related heteropenta­dienyl ligands and complexes, see: Boeré *et al.* (1998 ▶, 2004 ▶, 2005 ▶). For a description of the Cambridge Structural Database, see: Allen (2002 ▶).
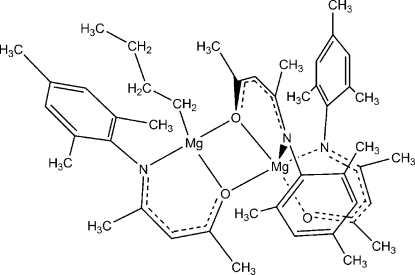


## Experimental

### 

#### Crystal data

[Mg_2_(C_4_H_9_)(C_14_H_18_NO)_3_]
*M* *_r_* = 754.61
Monoclinic, 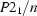

*a* = 20.016 (2) Å
*b* = 10.7515 (12) Å
*c* = 20.720 (2) Å
β = 94.154 (1)°
*V* = 4447.3 (8) Å^3^
*Z* = 4
Mo *K*α radiation
μ = 0.10 mm^−1^
*T* = 173 K
0.35 × 0.31 × 0.22 mm

#### Data collection

Bruker APEXII CCD area-detector diffractometer
Absorption correction: multi-scan (*SADABS*; Bruker, 2006 ▶) *T*_min_ = 0.906, *T*_max_ = 0.977
57968 measured reflections
9082 independent reflections
5810 reflections with *I* > 2σ(*I*)
*R*_int_ = 0.065

#### Refinement

*R*[*F* ^2^ > 2σ(*F* ^2^)] = 0.054
*wR*(*F* ^2^) = 0.166
*S* = 1.02
9082 reflections
503 parameters
H-atom parameters constrained
Δρ_max_ = 0.32 e Å^−3^
Δρ_min_ = −0.37 e Å^−3^

### 

Data collection: *APEX2* (Bruker, 2006 ▶); cell refinement: *SAINT-Plus* (Bruker, 2006 ▶); data reduction: *SAINT-Plus*; program(s) used to solve structure: *SHELXS97* (Sheldrick, 2008 ▶); program(s) used to refine structure: *SHELXTL* (Sheldrick, 2008 ▶); molecular graphics: *Mercury* (Macrae *et al.*, 2006 ▶); software used to prepare material for publication: *publCIF* (Westrip, 2009 ▶).

## Supplementary Material

Crystal structure: contains datablocks I, global. DOI: 10.1107/S1600536809033327/pv2200sup1.cif

Structure factors: contains datablocks I. DOI: 10.1107/S1600536809033327/pv2200Isup2.hkl

Additional supplementary materials:  crystallographic information; 3D view; checkCIF report

## Figures and Tables

**Table 1:** Comparative mean bond distances (Å) to Mg in 4, 5 and 6-coordinate ketiminate complexes

CN4 / Cmpd	(I)	(II)	(III)	(IV)		
O chelate		1.895 (2)	1.921 (5)	1.917 (2)		
O bridge	2.000 (2)					
N chelate		2.059 (1)	2.076 (6)	2.081 (8)		
N bridge*	2.107 (2)					
						
CN5 / Cmpd	(I)	(V)	(VI)	(VII)	(VIII)	
O chelate	1.951 (2)	1.972 (9)	1.945 (2)	1.954 (1)	1.952 (5)	
O bridge	2.06 (3)	2.025 (2)^#^			2.028 (5)	
N chelate	2.105 (2)	2.161 (2)	2.18 (3)	2.045 (2)	2.107 (5)	
N bridge*	2.153 (2)				2.125 (6)	
						
CN6 / Cmpd	(IX)	(X)	(XI)			
O chelate		2.018 (1)	2.007 (3)			
O bridge	2.075 (7)					
N chelate		2.162 (1)	2.307 (9)			
N bridge*	2.201 (5)					

Notes: compound (I) corresponds to the title compound and (II)–(XI) are defined in the supplementary material. (*) The ‘N bridge’ indicates a ketiminate N atom for ligands where the O donor is doubly-bridged between two Mg atoms. (#) Terminal rather than bridging ketiminate O atom.

**Fig. 1.**
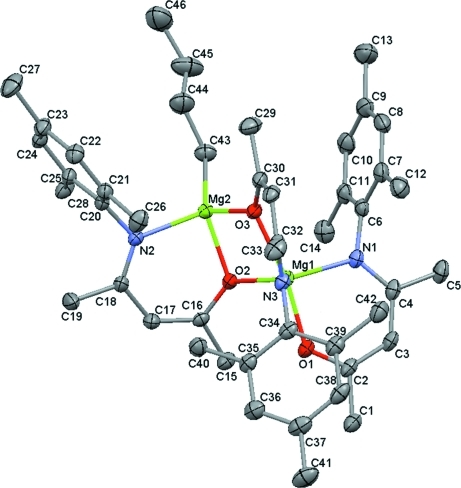
A view of (I), plotted with dispacement ellipsoids drawn at 30% probability level; H atoms are omited for clarity.

## supplementary crystallographic information

### Comment

The title compound (I) was prepared as part of our interest in heteropentadienyl ligands bearing bulky aryl substituents at nitrogen (and phosphorus) donor atoms (Boeré *et al.*, 1998, 2004, 2005). The structure (Fig. 1) of (I) contains two Mg atoms bridged by two µ_2_ oxygen atoms from two of the three ketiminate ligands, while the third ketiminate is strictly chelating to Mg1 which is thereby five coordinate. In place of this chelating ligand, Mg2 retains a butyl group originating in the di-*n*-butylmagnesium reagent used in its synthesis, and thus Mg2 is four coordinate. The geometry at Mg1 is close to trigonal bipyramidal with one chelating and one bridging oxygen in the axial positions and two chelating nitrogen and one bridging oxygen in the equatorial positions. The geometry at Mg2 is very distorted tetrahedral with the O2-Mg2-C43 angle at 131.0 (1)°. The terminal butyl group, unsurprisingly, has somewhat higher thermal motion parameters than the geometrically much more constrained ketiminate ligands. There are no significant inter-molecular contacts within the crystal lattice.

There are 14 previously reported Mg ketiminate complexes in the literature which display a wide variety of interesting structures (Refcodes: DAZDOY, GIKCIM, GUHQAB, KALJIR, REYYEA, REYYIE, REYYOK, TOQNAP, TOQNET, TOQNIX; Allen, 2002,). Bis(2-(2,6-Diisopropylphenylamino)pent-2-en-4-onato-*N*,*O*)-magnesium (II) (Lee, *et al.*, 2007); bis(4-*N*-(cyclohexylimino)pent-2-en-4-onato)-magnesium (III) (Ouattara, *et al.*, 2005) and bis(5-(2,2-dimethylhydrazido)-2,6-dimethyl-4-hepten-3-onato-*N*,*O*)-magnesium (IV) (Sedai, *et al.*, 2008) are 4-coordinate, distorted tetrahedral, with two chelating ketiminate ligands. Bis(2-(2,6-diisopropylphenylamino)pent-2-en-4-onato-*N*,*O*)-(2-(2,6-diisopropylphenylamino)pent-2-en-4-onato-*O*)-magnesium (V) is five coordinate with two chelating and one terminally-O bonded ketiminate ligands, while bis(2-(2,6-diisopropylphenylamino)pent-2-en-4-onato-*N*,*O*)-(pyridine-*N*)-magnesium (VI) is five coordinate with two chelating ketiminate and a terminal pyridine ligand (Lee, *et al.*, 2007). Bis(µ_2_-4-(2,2-dimethylhydrazido)-3-penten-2-onato-*N*,*O*,*O*)-bis(4-(2,2-dimethylhydrazido)-3-penten-2-onato-*N*,*O*)-di-magnesium (VII) is a dimer with five coordinate Mg atoms each bearing one chelating and one bridging ligand in which the oxygen atoms form a trapezoidal Mg_2_O_2 _central ring (Sedai, *et al.*, 2008). Bis(µ_2_-*N*,*N*'-ethylenebis(acetylacetoniminato-*O*,*O*,*O*',*N*,*N*'))-dimagnesium (VIII) is also an oxygen-bridged dimer, but it contains only two tetradentate diketiminate ligands which are linked by a CH_2_CH_2_ chain between the two imino donor atoms (Corazza, *et al.*). Hexakis(µ_2_-4-(*N*-n-butylimino)pentan-2-onato-*N*,*O*,*O*)-tri-magnesium (IX) is an interesting example of a six-coordinate Mg complex. Two terminal Mg atoms each have three chelating ketiminate ligands, while a central Mg is coordinated by all six O donor atoms in a bridging fashion (Matthews *et al.*, 2005). Bis(5-*N*-(*N*,*N*-dimethylaminopropyl)-2,2,7-trimethyl-3-octanonato)-magnesium (X) is six-coordinate octahedral by virtue of two ketiminate ligands with pendant CH_2_CH_2_NMe_2_ donors (Matthews *et al.*, 2000). Bis(5-(2,2-dimethylhydrazido)-2,6-dimethyl-4-hepten-3-onato-*N*,*O*)-transbis(4-*t*-butylpyridine)-magnesium (XI) bears two chelating ketiminate and two terminal pyridine donors, the latter in the axial position of the octahedral structure (Sedai, *et al.*, 2008). The remaining known structures are poorer comparisons to (I) because they each include an η-5 cyclopentadienyl ligand which leads to rather different geometries (Refcodes: TOQNOD, TOQNUY, TOQPAR; Allen, 2002,) or have no reported geometrical details (Tang *et al.*, 2007).

Mean Mg—L distances for the ketiminate donor groups for (I)-(XI) are presented in Table 1. The table shows some very interesting trends, within which the 4- and 5-coordinate distances found in (I) are squarely placed. For example, the Mg—O and Mg—N distances for chelating ketiminate ligands show distinct increases with increasing coordination number from four to six, despite the fact that a range of distances is found at each level. Notice also that in each case the Mg—O or Mg—N distances where the ligands participate in bridging are longer than those which are strictly chelating. This is true for the O donors which are µ_2_ coordinated to two Mg^2+^ ions each, but also for the N donors which bond to a single magnesium ion.

### Experimental

4-(*N*-2,4,6-trimethylphenylimino)pentane-2-one (0.317 g, 1.459 mmol) was dissolved in 12.5 ml dry heptane in a Schlenk tube and cooled in an ice/salt bath to 260 K and a heptane solution of dibutylmagnesium was added by syringe (0.8 ml of 1.0 *M*, 0.8 mmol). After completion of the addition, the reaction was allowed to warm to room temperature and stirred for a further hour. The heptane was removed by vacuum until solid started coming out of solution and then the residual mixture was heated till the solid re-dissolved in the remaining heptane. On placing in a freezer at 263 K, X-ray quality crystals of the title compound were obtained as large, yellow blocks.

### Refinement

All the non-H atoms were refined anisotropically and provided chemically reasonable positions without resorting to any restraints or constraints. H-atoms were included at geometrically idealized positions with C—H distances of 0.95 (aromatic), 0.99 (CH_2_) and 0.98 (CH_3_) Å and *U*_iso_ = 1.2 times *U*_eq_ of the C-atoms to which they are bonded. The model was refined to convergence. The highest residual peak was small (0.31 e^-^/Å^3^) but is located w.r.t. C44 of the butyl group in the correct location for a classic CH_2_ "elbow" disorder. Thermal coefficients for the terminal bultyl group are in any case higher than those for the backbone and mesityl group carbon atoms.

### Crystal data

**Table:**

[Mg_2_(C_4_H_9_)(C_14_H_18_NO)_3_]	*F*(000) = 1632
*M**_r_* = 754.61	*D*_x_ = 1.127 Mg m^−^^3^
Monoclinic, *P*2_1_/*n*	Mo *K*α radiation, λ = 0.71073 Å
Hall symbol: -P 2yn	Cell parameters from 8867 reflections
*a* = 20.016 (2) Å	θ = 2.3–23.5°
*b* = 10.7515 (12) Å	µ = 0.10 mm^−^^1^
*c* = 20.720 (2) Å	*T* = 173 K
β = 94.154 (1)°	Prism, yellow
*V* = 4447.3 (8) Å^3^	0.35 × 0.31 × 0.22 mm
*Z* = 4	

### Data collection

**Table:**

Bruker APEXII CCD area-detector diffractometer	9082 independent reflections
Radiation source: fine-focus sealed tube, Bruker D8	5810 reflections with *I* > 2σ(*I*)
graphite	*R*_int_ = 0.065
φ and ω scans	θ_max_ = 26.4°, θ_min_ = 2.0°
Absorption correction: multi-scan (*SADABS*; Bruker, 2006)	*h* = −25→24
*T*_min_ = 0.906, *T*_max_ = 0.977	*k* = −13→13
57968 measured reflections	*l* = −25→25

### Refinement

**Table:**

Refinement on *F*^2^	Primary atom site location: structure-invariant direct methods
Least-squares matrix: full	Secondary atom site location: difference Fourier map
*R*[*F*^2^ > 2σ(*F*^2^)] = 0.054	Hydrogen site location: inferred from neighbouring sites
*wR*(*F*^2^) = 0.166	H-atom parameters constrained
*S* = 1.02	*w* = 1/[σ^2^(*F*_o_^2^) + (0.0707*P*)^2^ + 3.119*P*] where *P* = (*F*_o_^2^ + 2*F*_c_^2^)/3
9082 reflections	(Δ/σ)_max_ < 0.001
503 parameters	Δρ_max_ = 0.32 e Å^−^^3^
0 restraints	Δρ_min_ = −0.37 e Å^−^^3^

### Special details

**Table:**

Geometry. All e.s.d.'s (except the e.s.d. in the dihedral angle between two l.s. planes) are estimated using the full covariance matrix. The cell e.s.d.'s are taken into account individually in the estimation of e.s.d.'s in distances, angles and torsion angles; correlations between e.s.d.'s in cell parameters are only used when they are defined by crystal symmetry. An approximate (isotropic) treatment of cell e.s.d.'s is used for estimating e.s.d.'s involving l.s. planes.
Refinement. Refinement of *F*^2^ against ALL reflections. The weighted *R*-factor *wR* and goodness of fit *S* are based on *F*^2^, conventional *R*-factors *R* are based on *F*, with *F* set to zero for negative *F*^2^. The threshold expression of *F*^2^ > σ(*F*^2^) is used only for calculating *R*-factors(gt) *etc*. and is not relevant to the choice of reflections for refinement. *R*-factors based on *F*^2^ are statistically about twice as large as those based on *F*, and *R*- factors based on ALL data will be even larger.

### Fractional atomic coordinates and isotropic or equivalent isotropic displacement parameters (Å^2^)

**Table:**

	*x*	*y*	*z*	*U*_iso_*/*U*_eq_	
Mg1	−0.00341 (4)	0.15436 (8)	0.20348 (4)	0.0312 (2)	
Mg2	0.11126 (4)	0.10955 (8)	0.31177 (4)	0.0325 (2)	
O1	−0.00970 (8)	0.17052 (16)	0.10943 (8)	0.0358 (4)	
O2	0.09942 (8)	0.12165 (15)	0.21528 (7)	0.0314 (4)	
O3	0.01501 (8)	0.16096 (16)	0.30108 (8)	0.0349 (4)	
N1	−0.06094 (10)	−0.0096 (2)	0.19363 (10)	0.0357 (5)	
N2	0.17489 (10)	0.26552 (19)	0.31215 (9)	0.0318 (5)	
N3	−0.05901 (10)	0.32324 (19)	0.21540 (10)	0.0350 (5)	
C1	−0.04882 (14)	0.1662 (3)	−0.00035 (13)	0.0472 (7)	
H15A	−0.0614	0.2543	0.0003	0.057*	
H15B	−0.0814	0.1205	−0.0288	0.057*	
H15C	−0.0042	0.1580	−0.0164	0.057*	
C2	−0.04800 (13)	0.1139 (3)	0.06704 (12)	0.0369 (6)	
C3	−0.08641 (13)	0.0111 (3)	0.07919 (13)	0.0413 (6)	
H17	−0.1137	−0.0209	0.0436	0.050*	
C4	−0.08961 (13)	−0.0517 (3)	0.13865 (13)	0.0407 (6)	
C5	−0.12758 (16)	−0.1737 (3)	0.13691 (16)	0.0546 (8)	
H19A	−0.0965	−0.2419	0.1485	0.066*	
H19B	−0.1484	−0.1876	0.0933	0.066*	
H19C	−0.1623	−0.1706	0.1678	0.066*	
C6	−0.06439 (13)	−0.0846 (2)	0.25060 (13)	0.0390 (6)	
C7	−0.11648 (14)	−0.0688 (3)	0.29138 (14)	0.0456 (7)	
C8	−0.11454 (17)	−0.1369 (3)	0.34859 (15)	0.0569 (9)	
H22	−0.1497	−0.1266	0.3765	0.068*	
C9	−0.06325 (18)	−0.2189 (3)	0.36633 (15)	0.0557 (8)	
C10	−0.01289 (17)	−0.2324 (3)	0.32513 (15)	0.0533 (8)	
H25	0.0227	−0.2883	0.3367	0.064*	
C11	−0.01210 (14)	−0.1677 (2)	0.26724 (14)	0.0435 (7)	
C12	−0.17251 (15)	0.0206 (3)	0.27396 (16)	0.0584 (8)	
H20A	−0.1541	0.1040	0.2683	0.070*	
H20B	−0.2033	0.0223	0.3087	0.070*	
H20C	−0.1968	−0.0060	0.2335	0.070*	
C13	−0.0619 (2)	−0.2901 (4)	0.42905 (17)	0.0822 (12)	
H24A	−0.0978	−0.2599	0.4548	0.099*	
H24B	−0.0185	−0.2778	0.4533	0.099*	
H24C	−0.0685	−0.3788	0.4199	0.099*	
C14	0.04347 (15)	−0.1869 (3)	0.22311 (16)	0.0524 (8)	
H27A	0.0254	−0.2258	0.1828	0.063*	
H27B	0.0778	−0.2409	0.2444	0.063*	
H27C	0.0634	−0.1064	0.2134	0.063*	
C15	0.14678 (13)	0.1038 (3)	0.11291 (11)	0.0393 (6)	
H14A	0.1485	0.0130	0.1166	0.047*	
H14B	0.1858	0.1333	0.0914	0.047*	
H14C	0.1057	0.1283	0.0875	0.047*	
C16	0.14727 (12)	0.1600 (2)	0.17895 (11)	0.0310 (5)	
C17	0.19433 (13)	0.2446 (2)	0.19922 (12)	0.0358 (6)	
H12	0.2254	0.2669	0.1687	0.043*	
C18	0.20325 (13)	0.3054 (2)	0.26160 (12)	0.0357 (6)	
C19	0.24825 (17)	0.4177 (3)	0.26465 (14)	0.0545 (8)	
H10A	0.2457	0.4592	0.3065	0.065*	
H10B	0.2338	0.4754	0.2298	0.065*	
H10C	0.2945	0.3916	0.2597	0.065*	
C20	0.18963 (12)	0.3257 (2)	0.37385 (11)	0.0325 (5)	
C21	0.14650 (13)	0.4164 (2)	0.39439 (12)	0.0377 (6)	
C22	0.15864 (14)	0.4660 (3)	0.45614 (13)	0.0438 (7)	
H6	0.1292	0.5278	0.4705	0.053*	
C23	0.21219 (14)	0.4278 (3)	0.49714 (13)	0.0457 (7)	
C24	0.25498 (13)	0.3395 (3)	0.47480 (12)	0.0412 (6)	
H3	0.2926	0.3139	0.5022	0.049*	
C25	0.24492 (12)	0.2870 (3)	0.41391 (12)	0.0362 (6)	
C26	0.08908 (15)	0.4633 (3)	0.35033 (15)	0.0528 (8)	
H9A	0.0542	0.4968	0.3763	0.063*	
H9B	0.0706	0.3947	0.3235	0.063*	
H9C	0.1050	0.5290	0.3224	0.063*	
C27	0.22286 (18)	0.4803 (4)	0.56497 (15)	0.0695 (10)	
H5A	0.2043	0.5645	0.5659	0.083*	
H5B	0.2709	0.4830	0.5779	0.083*	
H5C	0.2003	0.4272	0.5951	0.083*	
C28	0.29308 (14)	0.1916 (3)	0.39100 (13)	0.0459 (7)	
H1A	0.3263	0.1708	0.4265	0.055*	
H1B	0.3159	0.2255	0.3546	0.055*	
H1C	0.2684	0.1164	0.3771	0.055*	
C29	−0.01285 (16)	0.1521 (4)	0.41057 (14)	0.0615 (9)	
H33A	−0.0190	0.0617	0.4089	0.074*	
H33B	−0.0448	0.1887	0.4388	0.074*	
H33C	0.0329	0.1712	0.4277	0.074*	
C30	−0.02454 (13)	0.2056 (3)	0.34334 (12)	0.0394 (6)	
C31	−0.06998 (13)	0.2967 (3)	0.32936 (12)	0.0429 (7)	
H31	−0.0971	0.3200	0.3631	0.051*	
C32	−0.08170 (13)	0.3619 (3)	0.26931 (12)	0.0388 (6)	
C33	−0.12145 (16)	0.4816 (3)	0.27273 (15)	0.0557 (8)	
H29A	−0.1314	0.5143	0.2289	0.067*	
H29B	−0.0952	0.5428	0.2988	0.067*	
H29C	−0.1635	0.4649	0.2927	0.067*	
C34	−0.07408 (13)	0.3956 (2)	0.15778 (12)	0.0367 (6)	
C35	−0.02485 (14)	0.4728 (2)	0.13529 (12)	0.0388 (6)	
C36	−0.03917 (16)	0.5389 (3)	0.07850 (13)	0.0469 (7)	
H39	−0.0059	0.5924	0.0634	0.056*	
C37	−0.10038 (17)	0.5294 (3)	0.04308 (14)	0.0508 (8)	
C38	−0.14741 (15)	0.4488 (3)	0.06546 (14)	0.0493 (7)	
H41	−0.1892	0.4396	0.0412	0.059*	
C39	−0.13564 (14)	0.3807 (3)	0.12220 (13)	0.0423 (7)	
C40	0.04239 (15)	0.4845 (3)	0.17229 (14)	0.0487 (7)	
H38A	0.0363	0.5145	0.2161	0.058*	
H38B	0.0702	0.5434	0.1501	0.058*	
H38C	0.0644	0.4030	0.1747	0.058*	
C41	−0.1152 (2)	0.6065 (3)	−0.01740 (15)	0.0671 (10)	
H42A	−0.1449	0.5598	−0.0483	0.081*	
H42B	−0.0732	0.6252	−0.0370	0.081*	
H42C	−0.1370	0.6843	−0.0061	0.081*	
C42	−0.18706 (14)	0.2924 (3)	0.14444 (14)	0.0530 (8)	
H34A	−0.1680	0.2085	0.1479	0.064*	
H34B	−0.2262	0.2920	0.1132	0.064*	
H34C	−0.2006	0.3188	0.1868	0.064*	
C43	0.14225 (16)	−0.0420 (3)	0.37340 (13)	0.0501 (7)	
H46A	0.1081	−0.1077	0.3652	0.060*	
H46B	0.1840	−0.0742	0.3567	0.060*	
C44	0.1552 (2)	−0.0353 (4)	0.44479 (17)	0.0718 (10)	
H43A	0.1141	−0.0040	0.4632	0.086*	
H43B	0.1909	0.0269	0.4547	0.086*	
C45	0.1757 (2)	−0.1558 (4)	0.47962 (19)	0.0901 (14)	
H44A	0.1384	−0.2161	0.4733	0.108*	
H44B	0.2147	−0.1913	0.4592	0.108*	
C46	0.1937 (2)	−0.1421 (5)	0.5509 (2)	0.0985 (15)	
H45A	0.2319	−0.0854	0.5578	0.118*	
H45B	0.2056	−0.2236	0.5695	0.118*	
H45C	0.1553	−0.1083	0.5719	0.118*	

### Atomic displacement parameters (Å^2^)

**Table:**

	*U*^11^	*U*^22^	*U*^33^	*U*^12^	*U*^13^	*U*^23^
Mg1	0.0311 (4)	0.0316 (4)	0.0302 (4)	−0.0017 (3)	−0.0028 (3)	−0.0048 (3)
Mg2	0.0343 (5)	0.0333 (5)	0.0293 (4)	−0.0028 (4)	−0.0026 (3)	0.0028 (3)
O1	0.0366 (10)	0.0386 (10)	0.0311 (9)	0.0009 (8)	−0.0041 (7)	−0.0061 (8)
O2	0.0301 (9)	0.0350 (9)	0.0285 (8)	−0.0012 (7)	−0.0013 (7)	−0.0007 (7)
O3	0.0325 (9)	0.0434 (10)	0.0286 (9)	−0.0019 (8)	−0.0005 (7)	−0.0032 (8)
N1	0.0356 (12)	0.0339 (12)	0.0370 (12)	−0.0030 (9)	−0.0026 (9)	−0.0058 (9)
N2	0.0321 (11)	0.0355 (11)	0.0270 (10)	−0.0024 (9)	−0.0034 (8)	0.0007 (9)
N3	0.0357 (12)	0.0354 (12)	0.0335 (11)	0.0009 (9)	−0.0010 (9)	−0.0055 (9)
C1	0.0449 (16)	0.0589 (19)	0.0364 (14)	0.0018 (14)	−0.0057 (12)	−0.0068 (13)
C2	0.0337 (14)	0.0431 (15)	0.0329 (13)	0.0088 (12)	−0.0050 (11)	−0.0108 (12)
C3	0.0420 (15)	0.0424 (16)	0.0375 (14)	−0.0004 (13)	−0.0099 (12)	−0.0121 (12)
C4	0.0367 (15)	0.0362 (14)	0.0481 (16)	0.0003 (12)	−0.0048 (12)	−0.0128 (12)
C5	0.0550 (19)	0.0446 (18)	0.0621 (19)	−0.0103 (15)	−0.0109 (15)	−0.0128 (15)
C6	0.0400 (15)	0.0328 (14)	0.0431 (15)	−0.0107 (12)	−0.0039 (12)	−0.0042 (12)
C7	0.0418 (16)	0.0442 (16)	0.0502 (17)	−0.0124 (13)	−0.0011 (13)	−0.0056 (13)
C8	0.058 (2)	0.061 (2)	0.0531 (18)	−0.0261 (17)	0.0136 (15)	−0.0073 (16)
C9	0.068 (2)	0.0457 (18)	0.0517 (18)	−0.0188 (16)	−0.0081 (16)	0.0056 (14)
C10	0.059 (2)	0.0340 (15)	0.065 (2)	−0.0095 (14)	−0.0092 (16)	0.0050 (14)
C11	0.0464 (16)	0.0293 (14)	0.0534 (17)	−0.0093 (12)	−0.0064 (13)	−0.0007 (12)
C12	0.0431 (18)	0.072 (2)	0.061 (2)	−0.0050 (16)	0.0084 (15)	−0.0052 (17)
C13	0.110 (3)	0.076 (3)	0.059 (2)	−0.028 (2)	−0.005 (2)	0.0155 (19)
C14	0.0527 (18)	0.0359 (16)	0.068 (2)	0.0037 (14)	0.0009 (15)	−0.0006 (14)
C15	0.0408 (15)	0.0446 (16)	0.0319 (13)	0.0046 (12)	−0.0022 (11)	−0.0041 (12)
C16	0.0321 (13)	0.0331 (13)	0.0270 (12)	0.0057 (11)	−0.0026 (10)	0.0027 (10)
C17	0.0361 (14)	0.0407 (15)	0.0309 (13)	−0.0040 (12)	0.0042 (11)	0.0004 (11)
C18	0.0334 (14)	0.0400 (15)	0.0330 (13)	−0.0064 (11)	−0.0028 (11)	0.0016 (11)
C19	0.067 (2)	0.058 (2)	0.0394 (16)	−0.0294 (16)	0.0063 (14)	−0.0016 (14)
C20	0.0326 (13)	0.0351 (14)	0.0292 (12)	−0.0065 (11)	−0.0016 (10)	−0.0011 (10)
C21	0.0349 (14)	0.0377 (15)	0.0399 (14)	−0.0034 (12)	−0.0013 (11)	−0.0001 (12)
C22	0.0412 (16)	0.0452 (16)	0.0454 (16)	0.0019 (13)	0.0047 (12)	−0.0097 (13)
C23	0.0426 (16)	0.0569 (18)	0.0372 (14)	−0.0055 (14)	0.0001 (12)	−0.0114 (13)
C24	0.0348 (14)	0.0555 (18)	0.0321 (13)	−0.0023 (13)	−0.0058 (11)	−0.0022 (12)
C25	0.0308 (14)	0.0446 (15)	0.0329 (13)	−0.0059 (11)	−0.0005 (10)	−0.0028 (11)
C26	0.0526 (18)	0.0454 (17)	0.0582 (19)	0.0087 (14)	−0.0107 (15)	−0.0046 (15)
C27	0.064 (2)	0.095 (3)	0.0482 (19)	0.008 (2)	−0.0041 (16)	−0.0302 (19)
C28	0.0397 (16)	0.0580 (19)	0.0387 (15)	0.0073 (13)	−0.0073 (12)	−0.0070 (13)
C29	0.054 (2)	0.094 (3)	0.0363 (16)	0.0074 (18)	0.0052 (14)	0.0025 (16)
C30	0.0326 (14)	0.0543 (17)	0.0309 (13)	−0.0060 (13)	−0.0001 (11)	−0.0046 (12)
C31	0.0356 (15)	0.0601 (18)	0.0332 (13)	0.0003 (13)	0.0041 (11)	−0.0119 (13)
C32	0.0320 (14)	0.0450 (16)	0.0391 (14)	0.0007 (12)	0.0009 (11)	−0.0118 (12)
C33	0.0563 (19)	0.059 (2)	0.0514 (18)	0.0145 (16)	0.0032 (15)	−0.0171 (15)
C34	0.0428 (15)	0.0330 (14)	0.0337 (13)	0.0107 (12)	−0.0004 (11)	−0.0072 (11)
C35	0.0461 (16)	0.0309 (14)	0.0385 (14)	0.0047 (12)	−0.0029 (12)	−0.0050 (11)
C36	0.0615 (19)	0.0350 (15)	0.0435 (16)	0.0058 (14)	0.0000 (14)	−0.0020 (12)
C37	0.070 (2)	0.0405 (16)	0.0410 (16)	0.0172 (15)	−0.0047 (15)	−0.0028 (13)
C38	0.0499 (18)	0.0507 (18)	0.0446 (16)	0.0165 (15)	−0.0137 (13)	−0.0104 (14)
C39	0.0421 (16)	0.0418 (16)	0.0421 (15)	0.0116 (13)	−0.0029 (12)	−0.0102 (12)
C40	0.0519 (18)	0.0431 (16)	0.0496 (17)	−0.0064 (14)	−0.0056 (14)	0.0042 (13)
C41	0.089 (3)	0.060 (2)	0.0499 (19)	0.0203 (19)	−0.0115 (17)	0.0048 (16)
C42	0.0403 (16)	0.066 (2)	0.0516 (18)	0.0028 (15)	−0.0061 (13)	−0.0110 (15)
C43	0.0574 (19)	0.0479 (17)	0.0450 (16)	0.0050 (14)	0.0035 (14)	0.0086 (14)
C44	0.083 (3)	0.067 (2)	0.064 (2)	−0.001 (2)	−0.0053 (19)	0.0197 (19)
C45	0.100 (3)	0.101 (3)	0.071 (3)	0.027 (3)	0.020 (2)	0.038 (2)
C46	0.088 (3)	0.105 (4)	0.101 (3)	−0.009 (3)	−0.004 (3)	0.043 (3)

### Geometric parameters (Å, °)

**Table:**

Mg1—O1	1.9514 (18)	C20—C25	1.398 (3)
Mg1—O3	2.0304 (18)	C21—C22	1.391 (4)
Mg1—O2	2.0848 (18)	C21—C26	1.502 (4)
Mg1—N1	2.107 (2)	C22—C23	1.381 (4)
Mg1—N3	2.153 (2)	C22—H6	0.9500
Mg1—Mg2	3.1287 (11)	C23—C24	1.381 (4)
Mg2—O2	2.0005 (17)	C23—C27	1.515 (4)
Mg2—O3	2.0011 (19)	C24—C25	1.383 (3)
Mg2—N2	2.105 (2)	C24—H3	0.9500
Mg2—C43	2.134 (3)	C25—C28	1.508 (4)
O1—C2	1.278 (3)	C26—H9A	0.9800
O2—C16	1.326 (3)	C26—H9B	0.9800
O3—C30	1.313 (3)	C26—H9C	0.9800
N1—C4	1.318 (3)	C27—H5A	0.9800
N1—C6	1.435 (3)	C27—H5B	0.9800
N2—C18	1.300 (3)	C27—H5C	0.9800
N2—C20	1.444 (3)	C28—H1A	0.9800
N3—C32	1.304 (3)	C28—H1B	0.9800
N3—C34	1.438 (3)	C28—H1C	0.9800
C1—C2	1.504 (4)	C29—C30	1.509 (4)
C1—H15A	0.9800	C29—H33A	0.9800
C1—H15B	0.9800	C29—H33B	0.9800
C1—H15C	0.9800	C29—H33C	0.9800
C2—C3	1.380 (4)	C30—C31	1.354 (4)
C3—C4	1.410 (4)	C31—C32	1.433 (4)
C3—H17	0.9500	C31—H31	0.9500
C4—C5	1.515 (4)	C32—C33	1.517 (4)
C5—H19A	0.9800	C33—H29A	0.9800
C5—H19B	0.9800	C33—H29B	0.9800
C5—H19C	0.9800	C33—H29C	0.9800
C6—C7	1.399 (4)	C34—C35	1.394 (4)
C6—C11	1.400 (4)	C34—C39	1.399 (4)
C7—C8	1.391 (4)	C35—C36	1.387 (4)
C7—C12	1.501 (4)	C35—C40	1.505 (4)
C8—C9	1.383 (5)	C36—C37	1.386 (4)
C8—H22	0.9500	C36—H39	0.9500
C9—C10	1.375 (5)	C37—C38	1.384 (4)
C9—C13	1.507 (4)	C37—C41	1.514 (4)
C10—C11	1.388 (4)	C38—C39	1.390 (4)
C10—H25	0.9500	C38—H41	0.9500
C11—C14	1.504 (4)	C39—C42	1.497 (4)
C12—H20A	0.9800	C40—H38A	0.9800
C12—H20B	0.9800	C40—H38B	0.9800
C12—H20C	0.9800	C40—H38C	0.9800
C13—H24A	0.9800	C41—H42A	0.9800
C13—H24B	0.9800	C41—H42B	0.9800
C13—H24C	0.9800	C41—H42C	0.9800
C14—H27A	0.9800	C42—H34A	0.9800
C14—H27B	0.9800	C42—H34B	0.9800
C14—H27C	0.9800	C42—H34C	0.9800
C15—C16	1.495 (3)	C43—C44	1.485 (4)
C15—H14A	0.9800	C43—H46A	0.9900
C15—H14B	0.9800	C43—H46B	0.9900
C15—H14C	0.9800	C44—C45	1.525 (5)
C16—C17	1.354 (3)	C44—H43A	0.9900
C17—C18	1.448 (3)	C44—H43B	0.9900
C17—H12	0.9500	C45—C46	1.502 (5)
C18—C19	1.505 (4)	C45—H44A	0.9900
C19—H10A	0.9800	C45—H44B	0.9900
C19—H10B	0.9800	C46—H45A	0.9800
C19—H10C	0.9800	C46—H45B	0.9800
C20—C21	1.390 (4)	C46—H45C	0.9800
			
O1—Mg1—O3	170.21 (8)	H10B—C19—H10C	109.5
O1—Mg1—O2	97.11 (7)	C21—C20—C25	120.6 (2)
O3—Mg1—O2	77.45 (7)	C21—C20—N2	119.6 (2)
O1—Mg1—N1	89.00 (8)	C25—C20—N2	119.6 (2)
O3—Mg1—N1	100.61 (8)	C20—C21—C22	118.7 (2)
O2—Mg1—N1	113.46 (8)	C20—C21—C26	120.9 (2)
O1—Mg1—N3	92.46 (8)	C22—C21—C26	120.4 (3)
O3—Mg1—N3	85.09 (8)	C23—C22—C21	121.9 (3)
O2—Mg1—N3	130.01 (8)	C23—C22—H6	119.1
N1—Mg1—N3	115.66 (9)	C21—C22—H6	119.1
O1—Mg1—Mg2	136.12 (6)	C22—C23—C24	118.1 (2)
O3—Mg1—Mg2	38.76 (5)	C22—C23—C27	120.7 (3)
O2—Mg1—Mg2	39.04 (5)	C24—C23—C27	121.2 (3)
N1—Mg1—Mg2	107.94 (7)	C23—C24—C25	122.2 (3)
N3—Mg1—Mg2	113.96 (6)	C23—C24—H3	118.9
O2—Mg2—O3	80.09 (7)	C25—C24—H3	118.9
O2—Mg2—N2	88.84 (8)	C24—C25—C20	118.5 (2)
O3—Mg2—N2	110.96 (8)	C24—C25—C28	120.7 (2)
O2—Mg2—C43	130.99 (10)	C20—C25—C28	120.8 (2)
O3—Mg2—C43	120.85 (11)	C21—C26—H9A	109.5
N2—Mg2—C43	117.15 (11)	C21—C26—H9B	109.5
O2—Mg2—Mg1	41.02 (5)	H9A—C26—H9B	109.5
O3—Mg2—Mg1	39.44 (5)	C21—C26—H9C	109.5
N2—Mg2—Mg1	106.98 (6)	H9A—C26—H9C	109.5
C43—Mg2—Mg1	135.48 (10)	H9B—C26—H9C	109.5
C2—O1—Mg1	129.51 (17)	C23—C27—H5A	109.5
C16—O2—Mg2	123.32 (14)	C23—C27—H5B	109.5
C16—O2—Mg1	128.95 (14)	H5A—C27—H5B	109.5
Mg2—O2—Mg1	99.94 (8)	C23—C27—H5C	109.5
C30—O3—Mg2	130.48 (15)	H5A—C27—H5C	109.5
C30—O3—Mg1	127.03 (16)	H5B—C27—H5C	109.5
Mg2—O3—Mg1	101.80 (8)	C25—C28—H1A	109.5
C4—N1—C6	118.3 (2)	C25—C28—H1B	109.5
C4—N1—Mg1	124.86 (19)	H1A—C28—H1B	109.5
C6—N1—Mg1	116.66 (15)	C25—C28—H1C	109.5
C18—N2—C20	119.5 (2)	H1A—C28—H1C	109.5
C18—N2—Mg2	124.00 (17)	H1B—C28—H1C	109.5
C20—N2—Mg2	116.41 (15)	C30—C29—H33A	109.5
C32—N3—C34	118.3 (2)	C30—C29—H33B	109.5
C32—N3—Mg1	125.59 (18)	H33A—C29—H33B	109.5
C34—N3—Mg1	116.09 (15)	C30—C29—H33C	109.5
C2—C1—H15A	109.5	H33A—C29—H33C	109.5
C2—C1—H15B	109.5	H33B—C29—H33C	109.5
H15A—C1—H15B	109.5	O3—C30—C31	123.4 (2)
C2—C1—H15C	109.5	O3—C30—C29	114.6 (2)
H15A—C1—H15C	109.5	C31—C30—C29	122.0 (2)
H15B—C1—H15C	109.5	C30—C31—C32	127.2 (2)
O1—C2—C3	124.8 (2)	C30—C31—H31	116.4
O1—C2—C1	115.2 (2)	C32—C31—H31	116.4
C3—C2—C1	120.0 (2)	N3—C32—C31	122.9 (2)
C2—C3—C4	127.2 (2)	N3—C32—C33	121.8 (3)
C2—C3—H17	116.4	C31—C32—C33	115.3 (2)
C4—C3—H17	116.4	C32—C33—H29A	109.5
N1—C4—C3	123.1 (3)	C32—C33—H29B	109.5
N1—C4—C5	120.3 (3)	H29A—C33—H29B	109.5
C3—C4—C5	116.6 (2)	C32—C33—H29C	109.5
C4—C5—H19A	109.5	H29A—C33—H29C	109.5
C4—C5—H19B	109.5	H29B—C33—H29C	109.5
H19A—C5—H19B	109.5	C35—C34—C39	120.6 (2)
C4—C5—H19C	109.5	C35—C34—N3	119.3 (2)
H19A—C5—H19C	109.5	C39—C34—N3	120.0 (2)
H19B—C5—H19C	109.5	C36—C35—C34	118.8 (3)
C7—C6—C11	120.3 (3)	C36—C35—C40	120.8 (3)
C7—C6—N1	120.7 (2)	C34—C35—C40	120.5 (2)
C11—C6—N1	118.8 (2)	C37—C36—C35	122.1 (3)
C8—C7—C6	118.4 (3)	C37—C36—H39	118.9
C8—C7—C12	121.1 (3)	C35—C36—H39	118.9
C6—C7—C12	120.6 (3)	C38—C37—C36	117.7 (3)
C9—C8—C7	122.4 (3)	C38—C37—C41	121.5 (3)
C9—C8—H22	118.8	C36—C37—C41	120.7 (3)
C7—C8—H22	118.8	C37—C38—C39	122.4 (3)
C10—C9—C8	117.8 (3)	C37—C38—H41	118.8
C10—C9—C13	120.9 (3)	C39—C38—H41	118.8
C8—C9—C13	121.3 (3)	C38—C39—C34	118.3 (3)
C9—C10—C11	122.6 (3)	C38—C39—C42	121.1 (3)
C9—C10—H25	118.7	C34—C39—C42	120.6 (3)
C11—C10—H25	118.7	C35—C40—H38A	109.5
C10—C11—C6	118.5 (3)	C35—C40—H38B	109.5
C10—C11—C14	120.8 (3)	H38A—C40—H38B	109.5
C6—C11—C14	120.7 (3)	C35—C40—H38C	109.5
C7—C12—H20A	109.5	H38A—C40—H38C	109.5
C7—C12—H20B	109.5	H38B—C40—H38C	109.5
H20A—C12—H20B	109.5	C37—C41—H42A	109.5
C7—C12—H20C	109.5	C37—C41—H42B	109.5
H20A—C12—H20C	109.5	H42A—C41—H42B	109.5
H20B—C12—H20C	109.5	C37—C41—H42C	109.5
C9—C13—H24A	109.5	H42A—C41—H42C	109.5
C9—C13—H24B	109.5	H42B—C41—H42C	109.5
H24A—C13—H24B	109.5	C39—C42—H34A	109.5
C9—C13—H24C	109.5	C39—C42—H34B	109.5
H24A—C13—H24C	109.5	H34A—C42—H34B	109.5
H24B—C13—H24C	109.5	C39—C42—H34C	109.5
C11—C14—H27A	109.5	H34A—C42—H34C	109.5
C11—C14—H27B	109.5	H34B—C42—H34C	109.5
H27A—C14—H27B	109.5	C44—C43—Mg2	125.6 (2)
C11—C14—H27C	109.5	C44—C43—H46A	105.9
H27A—C14—H27C	109.5	Mg2—C43—H46A	105.9
H27B—C14—H27C	109.5	C44—C43—H46B	105.9
C16—C15—H14A	109.5	Mg2—C43—H46B	105.9
C16—C15—H14B	109.5	H46A—C43—H46B	106.2
H14A—C15—H14B	109.5	C43—C44—C45	116.9 (3)
C16—C15—H14C	109.5	C43—C44—H43A	108.1
H14A—C15—H14C	109.5	C45—C44—H43A	108.1
H14B—C15—H14C	109.5	C43—C44—H43B	108.1
O2—C16—C17	123.2 (2)	C45—C44—H43B	108.1
O2—C16—C15	115.9 (2)	H43A—C44—H43B	107.3
C17—C16—C15	120.9 (2)	C46—C45—C44	114.8 (4)
C16—C17—C18	128.2 (2)	C46—C45—H44A	108.6
C16—C17—H12	115.9	C44—C45—H44A	108.6
C18—C17—H12	115.9	C46—C45—H44B	108.6
N2—C18—C17	122.5 (2)	C44—C45—H44B	108.6
N2—C18—C19	121.7 (2)	H44A—C45—H44B	107.6
C17—C18—C19	115.7 (2)	C45—C46—H45A	109.5
C18—C19—H10A	109.5	C45—C46—H45B	109.5
C18—C19—H10B	109.5	H45A—C46—H45B	109.5
H10A—C19—H10B	109.5	C45—C46—H45C	109.5
C18—C19—H10C	109.5	H45A—C46—H45C	109.5
H10A—C19—H10C	109.5	H45B—C46—H45C	109.5
			
O1—Mg1—Mg2—O2	−2.68 (11)	C4—N1—C6—C7	−91.8 (3)
O3—Mg1—Mg2—O2	−170.01 (12)	Mg1—N1—C6—C7	92.8 (2)
N1—Mg1—Mg2—O2	105.38 (10)	C4—N1—C6—C11	92.7 (3)
N3—Mg1—Mg2—O2	−124.68 (10)	Mg1—N1—C6—C11	−82.7 (3)
O1—Mg1—Mg2—O3	167.33 (13)	C11—C6—C7—C8	0.4 (4)
O2—Mg1—Mg2—O3	170.01 (12)	N1—C6—C7—C8	−174.9 (2)
N1—Mg1—Mg2—O3	−84.61 (10)	C11—C6—C7—C12	179.7 (3)
N3—Mg1—Mg2—O3	45.33 (10)	N1—C6—C7—C12	4.3 (4)
O1—Mg1—Mg2—N2	64.77 (11)	C6—C7—C8—C9	0.0 (4)
O3—Mg1—Mg2—N2	−102.56 (11)	C12—C7—C8—C9	−179.3 (3)
O2—Mg1—Mg2—N2	67.46 (10)	C7—C8—C9—C10	−0.1 (5)
N1—Mg1—Mg2—N2	172.84 (9)	C7—C8—C9—C13	179.1 (3)
N3—Mg1—Mg2—N2	−57.23 (10)	C8—C9—C10—C11	−0.2 (4)
O1—Mg1—Mg2—C43	−107.55 (15)	C13—C9—C10—C11	−179.4 (3)
O3—Mg1—Mg2—C43	85.13 (15)	C9—C10—C11—C6	0.6 (4)
O2—Mg1—Mg2—C43	−104.86 (15)	C9—C10—C11—C14	−179.0 (3)
N1—Mg1—Mg2—C43	0.52 (15)	C7—C6—C11—C10	−0.7 (4)
N3—Mg1—Mg2—C43	130.45 (14)	N1—C6—C11—C10	174.8 (2)
O2—Mg1—O1—C2	126.9 (2)	C7—C6—C11—C14	178.9 (2)
N1—Mg1—O1—C2	13.4 (2)	N1—C6—C11—C14	−5.6 (4)
N3—Mg1—O1—C2	−102.3 (2)	Mg2—O2—C16—C17	−31.5 (3)
Mg2—Mg1—O1—C2	128.58 (19)	Mg1—O2—C16—C17	111.4 (2)
O3—Mg2—O2—C16	145.11 (18)	Mg2—O2—C16—C15	148.50 (17)
N2—Mg2—O2—C16	33.60 (18)	Mg1—O2—C16—C15	−68.6 (3)
C43—Mg2—O2—C16	−92.3 (2)	O2—C16—C17—C18	1.7 (4)
Mg1—Mg2—O2—C16	151.5 (2)	C15—C16—C17—C18	−178.3 (3)
O3—Mg2—O2—Mg1	−6.42 (8)	C20—N2—C18—C17	175.8 (2)
N2—Mg2—O2—Mg1	−117.93 (8)	Mg2—N2—C18—C17	−0.8 (4)
C43—Mg2—O2—Mg1	116.12 (14)	C20—N2—C18—C19	−3.5 (4)
O1—Mg1—O2—C16	28.9 (2)	Mg2—N2—C18—C19	179.9 (2)
O3—Mg1—O2—C16	−142.8 (2)	C16—C17—C18—N2	15.5 (4)
N1—Mg1—O2—C16	120.93 (19)	C16—C17—C18—C19	−165.1 (3)
N3—Mg1—O2—C16	−70.3 (2)	C18—N2—C20—C21	96.8 (3)
Mg2—Mg1—O2—C16	−149.2 (2)	Mg2—N2—C20—C21	−86.4 (2)
O1—Mg1—O2—Mg2	178.13 (8)	C18—N2—C20—C25	−87.0 (3)
O3—Mg1—O2—Mg2	6.39 (8)	Mg2—N2—C20—C25	89.8 (2)
N1—Mg1—O2—Mg2	−89.87 (9)	C25—C20—C21—C22	−1.4 (4)
N3—Mg1—O2—Mg2	78.85 (11)	N2—C20—C21—C22	174.8 (2)
O2—Mg2—O3—C30	−164.2 (2)	C25—C20—C21—C26	176.8 (3)
N2—Mg2—O3—C30	−79.3 (2)	N2—C20—C21—C26	−7.0 (4)
C43—Mg2—O3—C30	63.6 (3)	C20—C21—C22—C23	0.2 (4)
Mg1—Mg2—O3—C30	−170.8 (3)	C26—C21—C22—C23	−177.9 (3)
O2—Mg2—O3—Mg1	6.64 (8)	C21—C22—C23—C24	1.2 (4)
N2—Mg2—O3—Mg1	91.53 (9)	C21—C22—C23—C27	−177.9 (3)
C43—Mg2—O3—Mg1	−125.53 (12)	C22—C23—C24—C25	−1.4 (4)
O2—Mg1—O3—C30	164.9 (2)	C27—C23—C24—C25	177.6 (3)
N1—Mg1—O3—C30	−83.2 (2)	C23—C24—C25—C20	0.3 (4)
N3—Mg1—O3—C30	32.0 (2)	C23—C24—C25—C28	179.5 (3)
Mg2—Mg1—O3—C30	171.3 (2)	C21—C20—C25—C24	1.1 (4)
O2—Mg1—O3—Mg2	−6.43 (8)	N2—C20—C25—C24	−175.1 (2)
N1—Mg1—O3—Mg2	105.49 (9)	C21—C20—C25—C28	−178.0 (2)
N3—Mg1—O3—Mg2	−139.29 (9)	N2—C20—C25—C28	5.8 (4)
O1—Mg1—N1—C4	−6.5 (2)	Mg2—O3—C30—C31	140.9 (2)
O3—Mg1—N1—C4	175.4 (2)	Mg1—O3—C30—C31	−27.8 (4)
O2—Mg1—N1—C4	−103.8 (2)	Mg2—O3—C30—C29	−35.8 (3)
N3—Mg1—N1—C4	85.7 (2)	Mg1—O3—C30—C29	155.4 (2)
Mg2—Mg1—N1—C4	−145.3 (2)	O3—C30—C31—C32	−2.1 (5)
O1—Mg1—N1—C6	168.54 (18)	C29—C30—C31—C32	174.4 (3)
O3—Mg1—N1—C6	−9.59 (19)	C34—N3—C32—C31	179.6 (2)
O2—Mg1—N1—C6	71.22 (19)	Mg1—N3—C32—C31	2.3 (4)
N3—Mg1—N1—C6	−99.21 (19)	C34—N3—C32—C33	−1.6 (4)
Mg2—Mg1—N1—C6	29.77 (19)	Mg1—N3—C32—C33	−179.0 (2)
O2—Mg2—N2—C18	−18.0 (2)	C30—C31—C32—N3	14.9 (5)
O3—Mg2—N2—C18	−96.9 (2)	C30—C31—C32—C33	−163.9 (3)
C43—Mg2—N2—C18	118.7 (2)	C32—N3—C34—C35	102.3 (3)
Mg1—Mg2—N2—C18	−55.3 (2)	Mg1—N3—C34—C35	−80.1 (3)
O2—Mg2—N2—C20	165.39 (17)	C32—N3—C34—C39	−82.7 (3)
O3—Mg2—N2—C20	86.46 (17)	Mg1—N3—C34—C39	94.9 (2)
C43—Mg2—N2—C20	−58.0 (2)	C39—C34—C35—C36	2.9 (4)
Mg1—Mg2—N2—C20	128.07 (16)	N3—C34—C35—C36	177.9 (2)
O1—Mg1—N3—C32	170.3 (2)	C39—C34—C35—C40	−177.5 (2)
O3—Mg1—N3—C32	−19.2 (2)	N3—C34—C35—C40	−2.5 (4)
O2—Mg1—N3—C32	−88.3 (2)	C34—C35—C36—C37	−0.8 (4)
N1—Mg1—N3—C32	80.2 (2)	C40—C35—C36—C37	179.5 (3)
Mg2—Mg1—N3—C32	−45.7 (2)	C35—C36—C37—C38	−1.3 (4)
O1—Mg1—N3—C34	−7.11 (18)	C35—C36—C37—C41	177.8 (3)
O3—Mg1—N3—C34	163.39 (18)	C36—C37—C38—C39	1.5 (4)
O2—Mg1—N3—C34	94.30 (19)	C41—C37—C38—C39	−177.5 (3)
N1—Mg1—N3—C34	−97.18 (19)	C37—C38—C39—C34	0.4 (4)
Mg2—Mg1—N3—C34	136.85 (16)	C37—C38—C39—C42	−178.8 (3)
Mg1—O1—C2—C3	−11.8 (4)	C35—C34—C39—C38	−2.7 (4)
Mg1—O1—C2—C1	168.86 (17)	N3—C34—C39—C38	−177.6 (2)
O1—C2—C3—C4	−1.7 (4)	C35—C34—C39—C42	176.6 (2)
C1—C2—C3—C4	177.7 (3)	N3—C34—C39—C42	1.6 (4)
C6—N1—C4—C3	−176.7 (2)	O2—Mg2—C43—C44	173.9 (2)
Mg1—N1—C4—C3	−1.7 (4)	O3—Mg2—C43—C44	−81.4 (3)
C6—N1—C4—C5	2.5 (4)	N2—Mg2—C43—C44	59.4 (3)
Mg1—N1—C4—C5	177.51 (19)	Mg1—Mg2—C43—C44	−128.9 (3)
C2—C3—C4—N1	8.3 (4)	Mg2—C43—C44—C45	178.9 (3)
C2—C3—C4—C5	−170.9 (3)	C43—C44—C45—C46	175.2 (3)

### Table 1 Comparative mean bond distances (Å) to Mg in 4, 5 and 6-coordinate ketiminate complexes

**Table:**

CN4/Cmpd	(I)	(II)	(III)	(IV)		
O chelate		1.895 (2)	1.921 (5)	1.917 (2)		
O bridge	2.000 (2)					
N chelate		2.059 (1)	2.076 (6)	2.081 (8)		
N bridge*	2.107 (2)					
CN5 / Cmpd	(I)	(V)	(VI)	(VII)	(VIII)	
O chelate	1.951 (2)	1.972 (9)	1.945 (2)	1.954 (1)	1.952 (5)	
O bridge	2.06 (3)	2.025 (2)^#^			2.028 (5)	
N chelate	2.105 (2)	2.161 (2)	2.18 (3)	2.045 (2)	2.107 (5)	
N bridge*	2.153 (2)				2.125 (6)	
CN6/Cmpd	(IX)	(X)	(XI)			
O chelate		2.018 (1)	2.007 (3)			
O bridge	2.075 (7)					
N chelate		2.162 (1)	2.307 (9)			
N bridge*	2.201 (5)					

Notes: compound (I) corresponds to the title compound and (II)–(XI) are defined in the supplementary material. (*) The N bridge indicates a ketiminate N atom for ligands where the O donor is doubly-bridged between two Mg atoms. (#) Terminal rather than bridging ketiminate O atom.
